# A Comparative Analysis of Hybrid Deep Learning Models for Human Activity Recognition

**DOI:** 10.3390/s20195707

**Published:** 2020-10-07

**Authors:** Saedeh Abbaspour, Faranak Fotouhi, Ali Sedaghatbaf, Hossein Fotouhi, Maryam Vahabi, Maria Linden

**Affiliations:** 1 School of Innovation, Design, and Engineering, Mälardalen University, 72220 Västerås, Sweden; hossein.fotouhi@mdh.se (H.F.); maryam.vahabi@mdh.se (M.V.); maria.linden@mdh.se (M.L.); 2 Engineering Department, University of Qom, Qom 3716146611, Iran; 3 RISE Research Institutes of Sweden, 72212 Västerås, Sweden; ali.sedaghatbaf@ri.se; 4 ABB Corporate Research, 72226 Västerås, Sweden

**Keywords:** human activity recognition, deep learning, convolutional neural nets, long short-term memory, gated recurrent unit

## Abstract

Recent advances in artificial intelligence and machine learning (ML) led to effective methods and tools for analyzing the human behavior. Human Activity Recognition (HAR) is one of the fields that has seen an explosive research interest among the ML community due to its wide range of applications. HAR is one of the most helpful technology tools to support the elderly’s daily life and to help people suffering from cognitive disorders, Parkinson’s disease, dementia, etc. It is also very useful in areas such as transportation, robotics and sports. Deep learning (DL) is a branch of ML based on complex Artificial Neural Networks (ANNs) that has demonstrated a high level of accuracy and performance in HAR. Convolutional Neural Networks (CNNs) and Recurrent Neural Networks (RNNs) are two types of DL models widely used in the recent years to address the HAR problem. The purpose of this paper is to investigate the effectiveness of their integration in recognizing daily activities, e.g., walking. We analyze four hybrid models that integrate CNNs with four powerful RNNs, i.e., LSTMs, BiLSTMs, GRUs and BiGRUs. The outcomes of our experiments on the PAMAP2 dataset indicate that our proposed hybrid models achieve an outstanding level of performance with respect to several indicative measures, e.g., F-score, accuracy, sensitivity, and specificity.

## 1. Introduction

Human activity recognition (HAR) refers to the automatic identification of the physical activities of human beings. What makes automatic recognition of physical activities a challenging task is the diversity of the ways different people perform a specific activity. Furthermore, some activities may be performed simultaneously and there may be no cause-effect relationship between two consecutive activities [1]. Today, the ubiquity of sensors (e.g., accelerometers, gyroscopes, and magnetometers) and their availability in mobile platforms make it easy to measure or analyze different aspects of physical activities e.g., motion, location and direction. The data collected by sensors are widely used to develop solutions in several domains such as healthcare [2], security [3], robotics [4], transportation [5], sports [6], smart home [7] and smart city [8]. Furthermore, HAR is one of the most assisting technology tools to support the elderly’s daily life [9]. Due to the ever-increasing growth in the population of people aged more than 60 years old, the health care costs will also increase dramatically. This fact highlights the need for smart patient observation systems in which HAR plays a key role [10].

Generally, the sensors employed in HAR research can be categorized into three groups: (1) wearable sensors (e.g., gyroscopes, accelerometers, and IMU sensors) [11], (2) ambient sensors (e.g., cameras, GPSs and PIRs), and (3) sensors embedded in smartphones. Today, smartphones include a wide variety of sensors e.g., motion, location, and direction sensors, which have made them the focus of several research work in the HAR domain [12]. Even though ambient and smartphone sensors are more available and easier to use, they suffer from less flexibility and accuracy in comparison with wearable sensors. Furthermore, ambient sensors may violate privacy. However, plenty of the existing HAR techniques are based on the data collected by ambient and smartphone sensors [13,14,15,16]. PAMAP2 is the dataset studied in this paper. This dataset includes data of 18 daily activities collected by wearable sensors. Walking, running, cycling, and driving are some examples of the activities included in this dataset.

HAR is recognized as the problem of classifying the data collected by sensors into a set of well-known physical activities. For this reason, machine learning (ML) models such as Support Vector Machines (SVMs), Decision Trees (DTs) and Naive Bayes (NB) are typically used in the solutions proposed for this problem [16,17,18].

In recent years, deep learning (DL) models as a subclass of ML models attracted the attention of several HAR researchers [11,19,20,21]. DL is based on artificial neural networks (ANNs), and it is now the biggest trend in ML due to the ease of access to powerful processors and software frameworks. Compared to shallow ML models, automatic feature extraction is the most important advantage of DL models, which makes them also appealing to the HAR domain. In fact, picking the features appropriate for HAR usually requires a deep knowledge and experience. In shallow ML methods, heuristic feature selection techniques are employed which do not scale well for complex motion patterns [11,22,23]. In fact, they may work well for recognizing low-level activities, e.g., “walking”, but it is difficult to infer more complex activities such as “drinking tea” with them. Additionally, DL provides a better support for incremental learning with data streams [23]. Despite the benefits of DL-based methods, choosing the most suitable DL model(s) is challenging [22].

Convolutional Neural Networks (CNNs) are a widely applied class of ANNs including some convolution layers followed by some fully connected layers. CNNs have been investigated by several HAR work, and the results indicate the effectiveness of these models as presented in [11,22], which is due to their capability in capturing local dependency and scale invariance in signals [19]. In our previous work [24], we employed CNNs to classify the daily activities recorded in the WISDM (https://archive.ics.uci.edu/ml/machine-learning-databases/00507/) dataset.

Our analysis results admit the high accuracy of our models. However, we got better results when we combined the CNN model with a Recurrent Neural Network (RNN) called Long Short-Term Memory (LSTM). In fact, the combination led to a higher accuracy and a lower loss value. Through the combination of CNN and RNN models, we can use both the power of CNNs in feature extraction and the capability of RNNs in considering temporal dependencies among activities. As an extension of that work, we compare different hybrid DL models, consisting of CNNs and RNNs in this paper. Accuracy, sensitivity, specificity, precision, recall and f-score are the measures evaluated in this comparative analysis. In Summary, the contributions of this paper are the following:

- Modeling various combinations of CNNs and RNNs to process and classify human activity data,
- Evaluating well-known performance measures(i.e., *accuracy*, *precision*, *recall*, *f-score*, *sensitivity* and *specificity*) on the proposed hybrid models,
- Analyzing the effects of different hyper-parameters (e.g., number of layers, pooling size and kernel size) on the evaluation results.
- Comparing the performance of the proposed models with the ones reported on the same dataset.

The rest of the paper is organized as follows. Section 2 discusses the research contributions related to the scope of this paper. A brief introduction to the DL models used in this paper is presented in Section 3, and the proposed deep learning architecture is explained in Section 4. Section 5 shows the experimental setup and the analysis results, and finally, Section 6 concludes the paper and outlines the future directions.

## 2. State-of-the-Art

The research work related to the scope of this paper can be categorized into two groups: (1) Shallow ML-based, and (2) DL-based methods.

### 2.1. Shallow ML-Based Methods

Khan [25] uses Decision Trees (DTs) for classifying daily activities e.g., running, sitting and lying, based on the data collected by wearable accelerometers. As another work based on DTs, Fan et al. [26] use accelerometers embedded in smartphones to extract data related to daily activities e.g., walking and running. The collected data are then used to learn movement patterns using DTs. In [27], the authors present a system architecture based on Support Vector Machines (SVMs) for HAR. They validate two implementations of their architecture through three case studies, which involve data gathered by both wearable and smartphone sensors. As another approach based on SVMs, Anguita et al. use smartphones to gather daily activity data from elderly people and employ fixed-point arithmetic to improve the performance of SVMs [16]. HMMs are used in [28] to classify physical activities. In this approach, the authors combine shape and optical flow features extracted from the videos recorded by some cameras. To transform optical features to index sequences that can be processed by HMMs, they use K-Means clustering. Casale et al. [29] introduce a wearable sensor to collect acceleration data and use Random Forests (RFs) for activity classification. They achieve more than 90% accuracy in classifying daily activities.

In [30], the authors pay special attention to the noise in sensor data, and propose to use artificial hydrocarbon networks (AHNs) to develop a robust solution for the HAR problem. Literature reports affirm the robustness of AHNs to noisy data, and the results of the experiments on the PAMAP2 dataset indicate that AHNs are competitive to other well-known ML models e.g., KNNs, SVMs and NB with respect to several performance measures e.g., accuracy and sensitivity. Attal et al. [31] report experiments on different supervised and unsupervised ML methods for HAR. Their experimental results indicate that K-Nearest Neighbor (KNN) and Hidden Markov Model (HMM) have achieved the best performance among the supervised and unsupervised methods respectively. In another effort, the authors of [32] try various ML techniques e.g., KNNs, Logistic Regression (LR) and Naive Bayse (NB) for activity classification. The comparison of the outcomes indicates the superiority of KNNs. Both of the above studies focus on daily activities e.g., walking, and their main distinguishing factor is sensor type. In particular, in [32], the authors focus on data collected by smartphone sensors, whereas wearable sensors are investigated in [31].

### 2.2. DL-Based Methods

In recent years, several researchers proposed DL-based solutions for the HAR problem. Comprehensive surveys are provided in [9,23,33,34]. Among the DL models, CNNs attracted the attention of several HAR researchers. Ronao et al. [35] use a 1-Dimensional CNN to classify activity data recorded by smartphone sensors. They compare the performance of their proposed model with some shallow ML models e.g., SVMs and DTs. The results indicate that the CNN model is more accurate. Zebin et al. [20] use a 2-Dimensional CNN to classify six daily activities recorded from 12 volunteers. They compare their method with traditional ML methods with respect to both accuracy and computational overhead. The results indicate improvement with respect to both measures. Ha et al. [19] propose another method based on CNNs in which 2-Dimensional CNNs are employed for activity classification. In their work, they compare the performance of two CNN variants differing mainly based on the way weights are shared in each convolution layer. [11] presents another application of CNNs to HAR. Here, the purpose of the author is to investigate different sensor configurations and find the optimal sensor placement for lower-limb activities.

In [36], the authors apply different variants of RNNs (e.g., GRUs and LSTMs) to recognize daily activities and detect abnormal behavior of the elderly people suffering from dementia. They compare the performance of these models with shallow ML models. The comparison results indicate that RNNs outperform other ML models with respect to most of the evaluated measures (e.g., accuracy, precision and recall), and among the investigated RNN models, LSTMs performed slightly better. Singh et al. [37] use LSTMs to classify human activity data collected by smart-home sensors. They also compare LSTMs with CNNs and traditional ML moldels in [38]. Their evaluations indicate that LSTMs and CNNs outperform other ML models, and CNNs are much faster than LSTMs in training but less accurate. As future work, they propose to combine CNNs and LSTMs to take benefit of both. Noori et al. [39] present another HAR approach based on LSTMs. In that work, the changes in magnitude and angle of joints are extracted from video frames, and used to learn the sequence of motion features. Golestani et al. [40] introduce a wireless system for HAR. The main motivation behind this system is to establish a good trade-off between power consumption and classification accuracy. To achieve this goal, the system uses LSTMs for motion learning and magnetic induction for efficient physical movement detection.

### 2.3. Summary of Findings

Table 1 summarizes our findings on the related work. The criteria used for comparison include ML model, feature extraction method and sensor type. Generally, most of the reported studies indicate that DL models outperform the traditional ML models with respect to the HAR problem. However, each DL model has its own strengths and weaknesses, and there is no DL model that can address all the challenges in HAR. For example, CNNs are very powerful in extracting appropriate local features from sensor data. However, they are memory-less and ignore temporal dependencies between data records. On the other hand, RNNs are well-suited for problems in which temporal dependencies play an important role. In this paper, we report some experiments on various combinations of CNNs and RNNs and compare those combinations regarding the most important performance measures e.g., accuracy, precision, and recall.

## 3. Fundamentals

The biggest advantage of DL models is their capability of learning complex features from raw data. This eliminates the need of pre-knowledge and handcrafted feature extraction. In this section, we briefly introduce the DL models used in our experimental study.

### 3.1. CNNs

CNNs are deep learning models widely used in computer vision. The architecture of a CNN is very similar to that of a visual cortex in the human brain. Through some filters, CNNs are able to extract features (i.e., spatial and temporal dependencies) and distinguish the objects within the input image. The filters constitute the convolution layers, which are usually followed by some fully connected layers responsible for the classification task. Other than being good at learning features, through some pooling layers, CNNs can scale to massive datasets. In fact, the purpose of pooling layers is reducing the dimensionality of input data and also extracting dominant features, which are invariant with respect to rotation and position.

### 3.2. RNNs

RNNs are a type of ANNs, which include an internal memory. They are called recurrent, since the output computed for the current input is dependent on both the input and the past computation results. In fact, the current output is fed back to the network and it is used (together with the current input) to produce the next output. Structurally, RNNs consist of a chain of repeating modules. In standard RNNs, this repeating module has a simple structure, which consists of a tanh activation function being applied to newly computed output. The tanh function regulates the data values flowing throughout the whole RNN by keeping them between −1 and 1.

### 3.3. LSTMs and BiLSTMs

LSTMs are an extension of RNNs, which perform much better than standard RNNs when it comes to remembering dependencies for a long time. This capability is due to the structure of the repeating module in these networks. In LSTMs, the repeating module comprises four interacting layers. These layers include a layer called the cell state, together with three other layers called gates. Cell state acts as the RNN memory. Gates are ANN layers responsible for controlling the information added to/removed from the cell state. In other words, these gates allow more relevant information to flow to the cell state and prevent the flow of less relevant information.

Bidirectional LSTMs are an extension of LSTMs, which are trained once on data sequence itself and once on a reversed copy of it. In other words, BiLSTMs are a combination of two LSTMs, one fed with data sequence in normal time order and the other fed in reverse time order. The outputs of the two networks are then concatenated at each time step.

### 3.4. GRUs and BiGRUs

GRUs are another extension of RNNs that similar to LSTMs address the problem of short-term memory in RNNs. However, GRUs have two gates instead of three, and do not include the cell state. Therefore, GRUs are structurally simpler than LSTMs and train faster due to fewer tensor operations. However, this does not mean that they are superior to LSTMs. Which one is better depends on the use case. Similar to BiLSTMs, BiGRUs are a combination of two GRUs one working on normal time order and the other one on reverse time order.

## 4. Method

**Figure 1:**
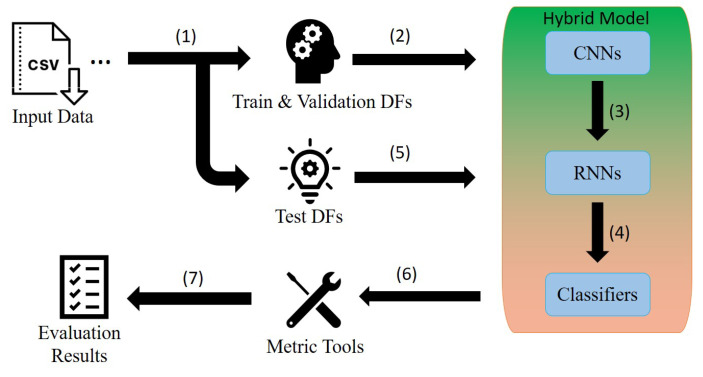
A schematic view of the analysis process.

In this section, we elaborate the steps of the analysis process. As illustrated in Figure 1, the process starts by (1) framing the input data into two groups;(a) train and validation and (b) test data frames. Then, the first group are (2) fed into the hybrid models for (3) extracting features and (4) learning temporal dependencies, and finally activity classification. The learned models are then (5) validated by the test data frames. In the next step, performance measures are evaluated on the classification results (6), and finally the hybrid models are compared based on the evaluation results (7). The above steps are explained in the following sections.

### 4.1. Dataset

In this paper, the train and test data are extracted from the PAMAP2 dataset [42]. This dataset includes data from nine subjects and includes 18 motion activities collected by three IMU sensors and a heart rate monitor. The activities include daily, household and sport activities and can be categorized into two groups: (1) stationary activities e.g., sitting, standing, lying, watching TV, and (2) dynamic activities e.g., walking, running, cycling, vacuum cleaning, and playing soccer. For data collection, each of the nine participants mounted three IMU sensors on their hand, chest and ankle. IMU has multiple sensors including accelerometer, gyroscope and magnetometer, which can be used to generate a time series of motion data. The sampling frequency of the IMU sensors was 100Hz and the sampling rate of the heart rate monitor was approximately 9 Hz. The heart rate data is not used in this paper.

Among the 18 activities in PAMAP2, only 12 activities are considered in this paper. These activities include walking (Wk), ironing (Ir), Nordic walking (NWk), standing (Sd), lying (Ly), sitting (St), vacuum cleaning (Vac), cycling (Cyc), going upstairs (AS), watching TV (WTV), going downstairs (DS), and running (Run). Among the data recorded for these activities, the data belonging to subjects 2 to 8 are selected for training and validation, and the rest for testing. Among the first group of data, 10,000 records are picked for validation.

### 4.2. DL Models

Each of the four DL models employed in the experiments reported in this paper integrates a CNN with a variant of RNNs. The variants include LSTMs, BiLSTMs, GRUs and BiGRUs. As emphasized in Section 2, CNNs are good at extracting local features by using appropriate filters, but they are weak at considering temporal dependencies among data records, which is the strength of RNNs.

**Figure 2:**
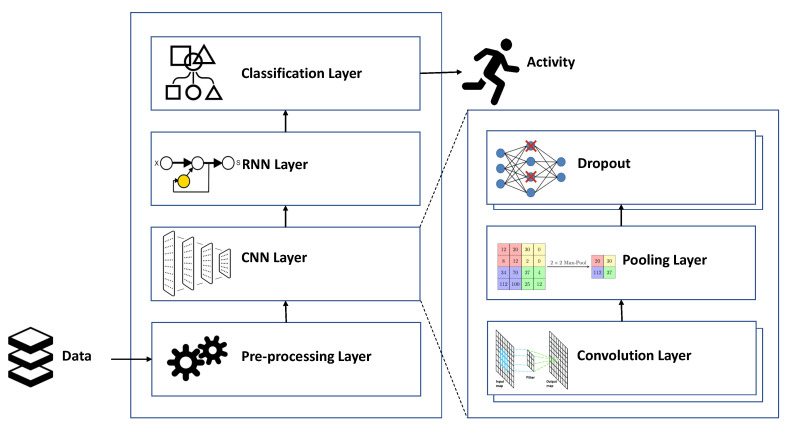
The architecture of the HAR system.

Figure 2 presents the architecture of the system implementing the hybrid models. Accordingly, the system includes a pre-processing module responsible for preparing the input data for the DL libraries. The input data is stored in nine files, each including the sensor data collected from a distinct individual. The pre-processing module loads the input data into matrices, which are then divided into time segments by a windowing technique called sliding windows. In this technique, data are divided into fixed-length windows. Event-based and activity-based windows are other types of windowing techniques typically used in ML processes [31]. In the event-based technique, specific events are located and used to partition data, whereas activity changes are the basis of the activity-based technique.

The partitioned data are then processed by the CNN model. As depicted in Figure 2, the CNN model consists of some 1-dimensional convolution layers each supplied with the ReLU activation function. Each convolution layer applies filters of size 512 each with a kernel of size 3 to extract feature maps from the input windows. To summarize the feature maps produced by the convolution layers and reduce the computational costs, a max-pooling layer with a pool of size 2 is also added to the CNN model. After reducing the size of the feature maps, we need to also reduce their dimensions to make them ready to be processed by the RNN models. To this end, the flatten layer converts the matrix representation of each feature map to a vector. For regularization and reducing the chance of over-fitting, some dropouts are added on top of the pooling layer. Using dropouts, the system ignores some neurons during the training phase. The ignored neurons are selected randomly and with a probability of 0.25.

After applying the dropout function, the output of the pooling layer is processed by a 100-neuron RNN layer, which models the temporal dynamics of the activation of the feature maps. The classification layer is the final layer of the system, which converts the class weights computed by the previous layers to probabilities. This layer includes two fully connected ANNs. The first network applies the ReLU activation function to its inputs. The neurons of this network are then dropped out with a probability of 0.5. The second network applies the Softmax activation function to the output of the dropout function. All the ANNs in the system use the Adam optimization algorithm to update weights and the cross entropy function to calculate loss. Adam optimizer is an efficient extension of stochastic gradient decent specifically designed for deep ANNs, and cross entropy is an information theoretic measure used to calculate the differences between two probability distributions and it is an efficient means to calculate classification error.

## 5. Experimental Results

As mentioned in Section 4, PAMAP2 is the dataset used in this research for learning and classifying human activities. The data in this dataset includes a lot of noise. Therefore, in the first step, we denoised the data using Wavelet transforms. To classify the denoised data, the architecture in Figure 2 is implemented using Python 3.7 in Google Colab. We also used the Tensorflow library, which provides efficient implementations for various kinds of DL models.

**Figure 3:**
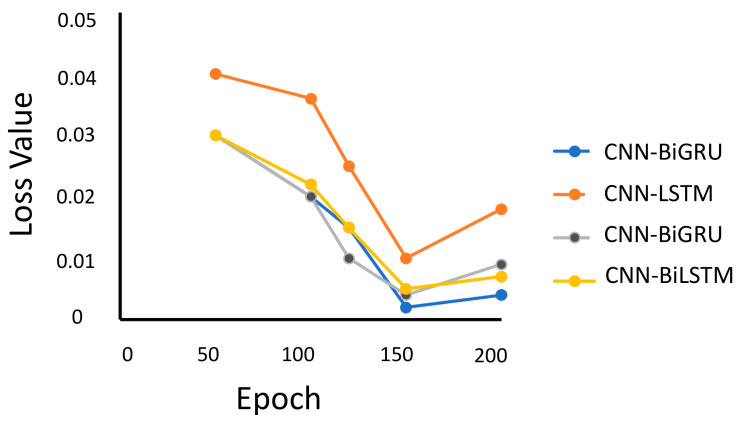
The loss value of the hybrid models.

To stabilize and speed up the training phase, batch normalization is used with a batch size of 50 data segments. The learning rate of the training is $10^{-3}$. Furthermore, sliding windows are used in this experiment to generate epochs with a duration of 3 s and 50% overlap. The hybrid models are configured to run over 50–200 epochs using ‘Sparse Categorical Cross Entropy’ as the loss function. Figure 3 shows the variation of loss value with respect to the number of epochs. Evidently, all of the four models have the least loss value at epoch 150, and CNN-BiGRU performs the best with this number of epochs.

### 5.1. Evaluation Results

To evaluate the classification results of the implemented hybrid models, the following quantitative metrics are used: *accuracy*, *precision*, *recall*, *F1-score*, *sensitivity* and *specificity*. *Accuracy* is the ratio of correctly classified data to the total data size. For each class in the classification output, *precision* indicates how many of data records predicted to belong to that class, are correctly classified. *Recall* specifies what proportion of the data belonging to that class are predicted correctly. *F1-score* is the harmonic mean of *precision* and *recall*. *Specificity* measures the proportion of the data predicted as not belonging to that class to the data that do not belong actually. Finally, *sensitivity* is measured in the same ways as *recall*. In fact, their difference is in their origination which is the statistics domain for *recall* and the information engineering domain for *sensitivity*. The above measures are evaluated for each activity in this experiment. Table 2 summarizes the evaluation results. Accordingly, all of the hybrid model have achieved more than 99% accuracy and specificity, and the CNN-BiGRU model has achieved the best performance with an average accuracy of 99.8%, and CNN-GRU is the worst with an average of 99.06%. These results also indicate the superiority of CNN-BiGRU with respect to the other performance measures.

### 5.2. Confusion Matrices

Table 3, Table 4, Table 5 and Table 6 present the confusion matrices of the four hybrid models. Comparing these matrices, it is apparent that CNN-BiGRU is the best at discriminating activities. All of the four models have achieved 100% accuracy with respect to *standing* and *lying* activities due to lack of movement in them. CNN-GRU and CNN-BiGRU can also perfectly distinguish *sitting* from other activities, and CNN-BiGRU is the only model that can also perfectly distinguish *walking*, while the other models might mistake this activity for other similar activities i.e., *Nordic walking* and *going upstairs*.

The confusion matrices of the Bi-directional models indicate that they are able to identify complex activities such as Nordic walking with at least 96% accuracy. Furthermore, CNN-GRU performs better than CNN-LSTM, and CNN-BiLSTM is superior to CNN-GRU in most of the cases. Last but not the least, we observe that all of the hybrid models have achieved a high level of accuracy in classifying very similar activities e.g., going downstairs (DS) and going upstairs (AS).

### 5.3. Comparison with Simple Models

To demonstrate the benefits of combining CNNs with RNNs, we compared the performance of the hybrid models to that of simple models which lack the CNN layers. The architecture and parameters of the simple models are exactly the same as the hybrid models except the CNN layer excluded from them. Accordingly, any difference in the performance outcomes is directly related to that architectural difference, rather than any specific optimization or customization. Table 7 presents the results of evaluating the same performance measures on the simple DL models. The results indicate that the CNN layer leads to performance improvement in all the models except the BiLSTM model whose performance does not change significantly.

### 5.4. Comparison with Previous Work

A list of past published ML/DL methods for HAR is presented in Table 8. All of these publications report experiments on the PAMAP2 dataset. *Accuracy* and *F1-score* are the metrics used for comparing them with the hybrid models introduced in this paper. We chose F1-score together with Accuracy, since both high rate of true positives/negatives and low rate of false positives/negatives are important performance indicators. Additionally, F1-score is suitable for datasets with imbalanced class distribution, which is the case for PAMAP2. Comparing the results in this table with the ones reported in Table 2, it is evident that the hybrid models proposed in this paper outperform the other models.

### 5.5. Discussion

In this experiment, the CNNs included in the hybrid models have two convolution layers. To investigate the impact of the number of convolution layers on the evaluation results, we repeated the experiment with CNNs consisting of 3 and 4 convolution layers. For the 3-layer case, we did not observe much difference in the evaluation results except for *specificity*, which raised up for three of the models i.e., CNN-LSTM, CNN-GRU and CNN-BiLSTM. Nevertheless, increasing the number of layers to four led to a slight improvement/degradation in the performance of the GRU-based/LSTM-based models. For example, the accuracy of the CNN-BiLSTM model decreased, while that of CNN-BiGRU increased. It is also possible to increase further the number of layers and possibly achieve better performance results. However, it would also slow down the training process. In fact, we need to establish a trade-off between the classification performance and the training overhead considering the availability of computational resources. In addition to the number of CNN layers, we analyzed the effect of changing the dropout probability and number of RNN neurons, but observed no difference in the performance results.

Our experiment confirms that the classification performance of the hybrid models is higher than the simple models. However, the training time required for the hybrid models is also higher. Furthermore, their performance is comparable to previous experiments on PAMAP2. Another interesting observation is the positive impact of batch normalization on the accuracy of the hybrid models. For example, if we remove this technique from the CNN-BiLSTM model, its F1-score would reduce to 93.38%.

## 6. Concluding Remarks and Future Directions

In this paper, we apply four hybrid DL models to the HAR problem. Each hybrid model integrates a CNN with a variant of RNNs. A well-known and publicly available dataset (i.e., PAMAP2) is used to evaluate the performance of the proposed hybrid models. The analysis results indicate a high level of accuracy for each model, which is higher than the accuracy achieved by using either CNNs or RNNs individually. In addition to accuracy, precision, recall, F-score, sensitivity, and specificity are other measures that are evaluated on the classification results. Overall, the results indicate that the models including Bi-directional RNNs perform better than the ones based on uni-directional RNNs. This outcome is reasonable due to the fact that in the former, the data are processed both from past to future and from future to past. However, this advantage comes with the cost of more computational time.

E-health is among the most prevalent application domains of HAR where the accuracy of predictions is of paramount importance. As future work, we plan to employ the proposed hybrid models in an end-to-end solution for reducing the fall risk of people suffering from Parkinson’s disease or other types of motion disorder. Furthermore, we intend to extend our studies to other datasets and DL models e.g., deep belief networks and deep Boltzmann machines.

## Figures and Tables

**Table 1:** Summary of findings in the related work

Ref.	Feature Extraction	ML Model(s)	Sensor Type
[26]	Manual	DTs	Smartphone
[25]	Manual	DTs	Wearable
[31]	Manual	KNNs, HMMs, SVMs, RFs	Wearable
[32]	Manual	KNNs, DTs, NB, LR	Smartphone
[16,27]	Manual	SVMs	Smartphone
[30]	Manual	AHNs	Wearable
[28]	Manual	SVMs, KNNs	Ambient
[29]	Manual	RFs	wearable
[35]	Automated	CNNs	Smartphone
[11,19,20]	Automated	CNNs	Wearable
[36]	Automated	VRNNs, GRUs, LSTMS	No sensor (synthesized data)
[37,39]	Automated	LSTMs	Ambient
[40,41]	Automated	LSTMs	Wearable
[38]	Automated	CNNs, LSTMs	Ambient
Current paper	Automated	CNN-LSTMs, CNN-BiLSTMs, CNN-GRUs, CNN-BiGRUs	Wearable

**Table 2:** Performance evaluation results for the hybrid models.

Metrics	CNN-LSTM	CNN-GRU	CNN-BiLSTM	CNN-BiGRU
Accuracy	99.57%	99.06%	99.65%	99.8%
Precision	93.16%	92.6%	94.55%	95.12%
Recall	93.16%	92.6%	94.55%	95.12%
F1-score	93.16%	92.6%	94.55%	95.12%
Sensitivity	93.16%	92.6%	94.55%	95.12%
Specificity	99.31%	99.29%	99.47%	99.55%

**Table 3:** Confusion matrix of the CNN-LSTM model.

Activity (%)	Wk	Ir	NWk	Sd	Ly	St	Vac	Cyc	AS	WTV	DS	Run
Wk	97.3	0	2.7	0	0	0	0	0	0.8	0	0	0
Ir	0	95.4	0	0	0	0	3	1.6	0	0	0	0
NWk	6	0	94	0	0	0	0	0	0	0	0	0
Sd	0	0	0	100	0	0	0	0	0	0	0	0
Ly	0	0	0	0	100	0	0	0	0	0	0	0
St	0	0	0	0	0	97	0	0	0	3	0	0
Vac	0	3.7	0	0	0	0	96.3	0	0	0	0	0
Cyc	1.8	0	0	0	0	0	0	98.2	0	0	0	0
AS	2.5	0	0	0	0	0	0	0	97.5	0	0	0
WTV	0	0	0	0	0	2.7	0	0	0	97.3	0	0
DS	4.3	0	0	0	0	0	0	0	0	0	95.7	0
Run	0	0	0	0	0	0	0	3.7	0	0	0	96.3

**Table 4:** Confusion matrix of the CNN-BiLSTM model.

Activity (%)	Wk	Ir	NWk	Sd	Ly	St	Vac	Cyc	AS	WTV	DS	Run
Wk	98.8	0	1	0	0	0	0	0	1.2	0	0	0
Ir	0	97.4	0	0	0	0	2.6	0	0	0	0	0
NWk	4	0	96	0	0	0	0	0	0	0	0	0
Sd	0	0	0	100	0	0	0	0	0	0	0	0
Ly	0	0	0	0	100	0	0	0	0	0	0	0
St	0	0	0	0	0	99	0	0	0	1	0	0
Vac	0	2	0	0	0	0	97.5	0	0	0	0	0
Cyc	0.02	0	0	0	0	0	0	99.98	0	0	0	0
AS	1.3	0	0	0	0	0	0	0	98.7	0	0	0
WTV	0	0	0	0	0	1.2	0	0	0	98.8	0	0
DS	1.5	0	0	0	0	0	0	0	0	0	98.5	0
Run	0	0	0	0	0	0	0	1.3	0	0	0	98.7

**Table 5:** Confusion matrix of the CNN-GRU model.

Activity (%)	Wk	Ir	NWk	Sd	Ly	St	Vac	Cyc	AS	WTV	DS	Run
Wk	98	0	2	0	0	0	0	0	0.8	0	0	0
Ir	0	96.7	0	0	0	0	2	1.3	0	0	0	0
NWk	5.2	0	94.8	0	0	0	0	0	0	0	0	0
Sd	0	0	0	100	0	0	0	0	0	0	0	0
Ly	0	0	0	0	100	0	0	0	0	0	0	0
St	0	0	0	0	0	100	0	0	0	0	0	0
Vac	0	2.2	0	0	0	0	97.8	0	0	0	0	0
Cyc	1	0	0	0	0	0	0	98.1	0	0	0	0.9
AS	2.1	0	0	0	0	0	0	0	97.9	0	0	0
WTV	0	0	0	0	0	2.7	0	0	0	97.3	0	0
DS	3	0	0	0	0	0	0	0	0	0	97	0
Run	0	0	0	0	0	0	0	3.6	0	0	0	96.4

**Table 6:** Confusion matrix of the CNN-BiGRU model.

Activity (%)	Wk	Ir	NWk	Sd	Ly	St	Vac	Cyc	AS	WTV	DS	Run
Wk	100	0	0	0	0	0	0	0	0	0	0	0
Ir	0	98.4	0	0	0	0	1.2	0.4	0	0	0	0
NWk	1	0	99	0	0	0	0	0	0	0	0	0
Sd	0	0	0	100	0	0	0	0	0	0	0	0
Ly	0	0	0	0	100	0	0	0	0	0	0	0
St	0	0	0	0	0	100	0	0	0	0	0	0
Vac	0	1.4	0.6	0	0	0	98	0	0	0	0	0
Cyc	0.5	0	0	0	0	0	0	98	0	0	0	1.5
AS	1.6	0	0	0	0	0	0	0	98.4	0	0	0
WTV	0	0	0	0	0	2	0	0	0	98	0	0
DS	1	0	0.2	0	0	0	0	0	0	0	98.8	0
Run	0	0	1	0	0	0	0	0	0	0	0	99

**Table 7:** Performance evaluation results for the simple models.

Metrics	LSTM	GRU	BiLSTM	BiGRU
Accuracy	98.78%	97.88%	99.53%	99.57%
Precision	91.21%	90.4%	94.47%	93.98%
Recall	91.21%	90.4%	94.47%	93.98%
F1-score	91.21%	90.4%	94.47%	93.98%
Sensitivity	91.21%	90.4%	94.47%	93.98%
Specificity	99.17%	99.09%	99.48%	99.45%

**Table 8:** Classification methods applied to the PAMAP2 dataset.

Method	Description	Accuracy	F1-Score
CNNs [43]	CCNs are used for feature extraction from acceleration time series.	91%	91.16%
LSTMs [43]	The performance of LSTMs for real-time HAR is analyzed and compared with some other DL/ML models.	85.86%	85.34%
LSTMs [44]	Temporal and sensor attentions are added to LSTMs to improve their performance for HAR.	-	89.96%
BiLSTMs [43]	BiLSTMs are applied to the real-time HAR domain.	89.52%	89.4%
CNN-LSTMs [45]	The HAR performance of CNNs and that of CNN-LSTMs are compared.	88.68%	88.98%
SVMs [43]	The application of SVMs to real-time HAR is investigated and their performance is compared to some other ML/DL models.	84.07%	83.76%
SVMs [46]	class-based decision fusion is used for effective combination of sensor data.	-	82.32%
KNNs [47]	A feature extraction technique is proposed for accelerometer data recorded by sensors in smart devices.	-	91.1%
